# Porous polydimethylsiloxane films with specific surface wettability but distinct regular physical structures fabricated by 3D printing

**DOI:** 10.3389/fbioe.2023.1272565

**Published:** 2023-09-21

**Authors:** Zhoukun He, Na Wang, Linpeng Mu, Zhuo Wang, Jie Su, Yikun Chen, Mingdong Luo, Ya Wu, Xiaorong Lan, Jiayan Mao

**Affiliations:** ^1^ School of Mechanical Engineering, Chengdu University, Chengdu, China; ^2^ Institute for Advanced Study, Research Center of Composites and Surface and Interface Engineering, Chengdu University, Chengdu, China; ^3^ Luzhou Key Laboratory of Oral and Maxillofacial Reconstruction and Regeneration, The Affiliated Stomatological Hospital of Southwest Medical University, Luzhou, China; ^4^ Institute of Stomatology, Southwest Medical University, Luzhou, China; ^5^ Department of Vascular Surgery, The Affiliated Hospital of Southwest Medical University, Luzhou, China; ^6^ Metabolic Vascular Disease Key Laboratory of Sichuan Province, The Affiliated Hospital of Southwest Medical University, Luzhou, China; ^7^ Department of Nephrology, Sichuan Provincial People’s Hospital, University of Electronic Science and Technology of China, Chengdu, China; ^8^ Chinese Academy of Sciences Sichuan Translational Medicine Research Hospital, Chengdu, China

**Keywords:** 3D printing, PDMS, superhydrophobic, surface wettability, anisotropic

## Abstract

Porous polydimethylsiloxane (PDMS) films with special surface wettability have potential applications in the biomedical, environmental, and structural mechanical fields. However, preparing porous PDMS films with a regular surface pattern using conventional methods, such as chemical foaming or physical pore formation, is challenging. In this study, porous PDMS films with a regular surface pattern are designed and prepared using 3D printing to ensure the formation of controllable and regular physical structures. First, the effect of the surface wettability of glass substrates with different surface energies (commercial hydrophilic glass and hydrophobic glass (F-glass) obtained by treating regular glass with 1*H*,1*H*,2*H*,2*H*-perfluorooctyl-trichlorosilane) on the structural characteristics of the 3D printed PDMS filaments is investigated systematically. Additionally, the effect of the printing speed and the surface wettability of the glass substrate on the PDMS filament morphology is investigated synchronously. Next, using the F-glass substrate and an optimized printing speed, the effects of the number of printed layers on both the morphologies of the individual PDMS filaments and porous PDMS films, and the surface wettability of the films are studied. This study reveals that regularly patterned porous PDMS films with distinct structural designs but the same controllable surface wettability, such as anisotropic surface wettability and superhydrophobicity, can be easily fabricated through 3D printing. This study provides a new method for fabricating porous PDMS films with a specific surface wettability, which can potentially expand the application of porous PDMS films.

## 1 Introduction

Surface wettability, a typical material property that occurs commonly in nature, is gaining increasing attention for both scientific researches and biomedical applications in antifouling materials (Xie et al., 2019; He et al., 2021a; He et al., 2021b; Chang et al., 2022; Eloffy et al., 2022; Rasitha et al., 2022; Seli et al., 2022; Selim et al., 2022; He et al., 2023a; He et al., 2023b; He et al., 2023c), blood contacting materials (Yang et al., 2020; Li et al., 2021; Li et al., 2022a; Li et al., 2022b), *etc.* (Wu et al., 2018; Li et al., 2020a; Wang et al., 2020; Guo et al., 2021; Han and Gong, 2021; Saji, 2021; Zhang et al., 2021; Zhu et al., 2021; Al-Bishari et al., 2022; Wang et al., 2022a; Luo et al., 2022; Yao et al., 2022; Yu et al., 2022; Zhang et al., 2022; Gresham and Neto, 2023; Li et al., 2023; Liao et al., 2023; Pan et al., 2023). Surfaces can be categorized according to the water contact angle (WCA), as hydrophilic (for WCA <90°) or hydrophobic (for WCA >90°) (Li et al., 2020b; Yang et al., 2022; Zhao et al., 2022). In particular, a surface is considered superhydrophilic or superhydrophobic when the WCA is lower than 10° or higher than 150°, respectively (Ji et al., 2021; Cao et al., 2022; He et al., 2022). Furthermore, according to the anisotropy of the WCA, the surface wettability can be either isotropic or anisotropic surface wettability (ASW). Various surface wettability phenomena occur in nature, such as the isotropic superhydrophobicity of lotus leaves (Zhang et al., 2009) and rose petals (Feng et al., 2008), isotropic superhydrophilicity of snail shells (Jiang et al., 2015), anisotropic superhydrophobicity of rice leaves and butterfly wings, and slippery surface wettability of ice (Rosenberg, 2005), pitcher plants (Bohn and Federle, 2004), and loach skin (Seo et al., 2021). Inspired by nature, biomimetic materials with special surface wettability characteristics have been designed and fabricated by controlling the surface morphology and chemical composition following the principles of Young’s equation (Young, 1805), and the Wenzel (Wenzel, 1936) and Cassie–Baxter models (Cassie and Baxter, 1944).

ASW has extensive applications, including in microfluidic devices (Zhang et al., 2015), directional self-cleaning surfaces (Zhao and Law, 2012), and printing industries (Xia et al., 2012). It is typically realized using anisotropic chemical compositions and/or anisotropic physical structures to vary the surface wettability characteristics in different directions. Therefore, designing ASW is significantly more complicated than designing isotropic surface wettability. Thus, most reports have focused primarily on isotropic surface wettability (He et al., 2011; He et al., 2012). Moreover, several of the reported methods, such as vacuum ultraviolet photolithography (Mor et al., 2005), optical lithography (Bliznyuk et al., 2009), interference lithography (Wu et al., 2010), unidirectional rubbing (Kusumaatmaja et al., 2008), electrospinning (Wu et al., 2008), and replica molding (Zhang et al., 2011; Xu et al., 2015), can be used to fabricate only two-dimensional (planar) or simple three-dimensional (3D) structures. Therefore, the fabrication of complex 3D porous surfaces with ASW using controllable and regular physical structures remains a formidable challenge. For example, the fabrication of such structures usually requires complicated photomasks or molds, or special experimental conditions. More importantly, different types of masks or molds are required to realize different ASW characteristics, thus resulting in additional costs and difficulty in fabricating the masks or molds with designed structural characteristics. Moreover, the distortion of anisotropic physical structures produced using the replica molding method cannot be avoided. Therefore, the fabrication of complex 3D porous surfaces with ASW using controllable and regular physical structures is a significant challenge.

Recently, the remarkable advantages of additive manufacturing (3D printing) in the fabrication of complicated structures have been demonstrated by numerous studies (He et al., 2017; Mallakpour et al., 2021a; Mallakpour et al., 2021b; Mallakpour et al., 2022; Marovič et al., 2023). Porous and complex physical structures can be easily fabricated via 3D printing because they can be designed and printed in a layerwise manner using computer programs. This process saves time and enables easy modification of the process parameters as no molding is required. Polydimethylsiloxane (PDMS) as one typical polymer material has been extensively researched for various applications (Song et al., 2021; Wang et al., 2022b; Ghahramani et al., 2022; Shinde et al., 2023). Previously, we fabricated substrates with ASW and superhydrophobicity by 3D printing parallel filaments of PDMS to obtain porous, complex, and controllable physical structures; subsequently, we demonstrated their potential application in air-breathable waterproofing, water-repellent floating carriers, and no-loss liquid transfer (He et al., 2017). The ASW and superhydrophobicity of porous PDMS structures could be tailored well by controlling the printing speed and filament spacing.

In this study, we focused on the effects of the wettability of the substrate on which the designed structures are printed, the number of printed PDMS layers, and the relative architecture of the different layers in the PDMS film on the final surface wettability and physical structures of the printed PDMS film. ASW with a difference in WCA of approximately 30° between the perpendicular and parallel directions of the porous PDMS film was achieved by implementing anisotropic physical structures without any special anisotropic chemical treatment and/or complicated anisotropic molding process. Briefly, anisotropic physical structures based on macroscale PDMS filaments were fabricated using a one-step 3D-printing process, and Wenzel-state superhydrophobicity with a WCA of approximately 156° in the perpendicular direction was obtained. Interestingly, specific ASW and superhydrophobicity could be obtained with the same surface chemical composition but distinct regular physical structures fabricated by 3D printing. This study demonstrates that porous PDMS films with a specific surface wettability but distinct regular physical structures can be obtained. Further, it provides a new design concept for fabricating porous PDMS films with a specific surface wettability and expands the application of such films in the biomedical, environmental, and structural mechanical fields.

## 2 Materials and methods

### 2.1 Materials

A polydimethylsiloxane adhesive (SE1700) with a curing agent was purchased from Dow Corning, United States 3-Butyn-1-ol (97%) and 1*H*,1*H*,2*H*,2*H*-perfluorooctyl-trichlorosilane (CF_3_(CF_2_)_5_CH_2_CH_2_SiCl_3_; PFTS, 97%) were purchased from Sigma-Aldrich. Toluene and absolute ethanol were purchased from the Chengdu Kelong Chemical Company, China.

### 2.2 Sample preparation

The PDMS ink preparation and 3D-printing processes were conducted as described in our previous report, with slight modifications (He et al., 2017). Briefly, 30 g of PDMS and 3 g of the curing agent were pre-mixed with 0.3 g of 3-butyn-1-ol for 30 min, and the resulting mixture was degassed for 2 h at room temperature. This mixture was then loaded into a syringe barrel and centrifuged at 8,000 rpm for 20 min before being used in 3D printing. To investigate the effect of the surface wettability of the glass substrate on the morphology and surface wettability of the printed PDMS film, hydrophilic (commercial glass) and hydrophobic glass substrates were used. The hydrophobic glass (F-Glass) substrate was prepared by treating the hydrophilic glass with 1.0 vol% PFTS in toluene for 1 min, as reported previously (He et al., 2011). The PDMS ink was extruded from a micronozzle with an inner diameter of 150 μm using a 3D printer. For printing PDMS filaments on the glass substrates, the micronozzle was moved along the X- and *Y*-axes at a programmed printing speed with a presupposed center-to-center filament spacing (FS). To print multilayer filaments for obtaining a porous PDMS film (area, 10 mm × 10 mm), the microneedle was shifted vertically (along the *Z*-axis) by 0.25 mm after the first layer was printed, and the subsequent layer was printed under the same conditions. Finally, the film samples were thermally cured at 120°C for 1 h and were peeled off the substrate for further characterization.

### 2.3 Characterization

#### 2.3.1 Surface wettability

Static WCAs were measured using a Krüss DSA100 (Germany) contact angle goniometer using 5 μL droplets of deionized water at ambient temperature. Five measurements were conducted at five different locations on the PDMS surface, and the results were averaged. The WCA of the printed filaments was measured in both the perpendicular (θ_**⊥**_) and parallel (θ_**∥**_) directions, and the difference (Δθ) between θ_**⊥**_ and θ_**∥**_ was used to evaluate the ASW of the porous PDMS.

#### 2.3.2 Physical structures

The surface morphology and average surface roughness (*R*a) of the PDMS film were characterized using a Seiko SPI4000 (Japan) atomic force microscope (AFM) in the tapping mode. The physical structures of the filaments and porous PDMS specimens were imaged using a Nikon LV100D optical microscope (Tokyo, Japan). The experimental PDMS filament diameter (FD) and FS in the x- and y-directions were statistically averaged based on measurements at five different locations.

## 3 Results and discussion

### 3.1 Effect of the wettability of the glass substrate on the PDMS filament

**FIGURE 1:**
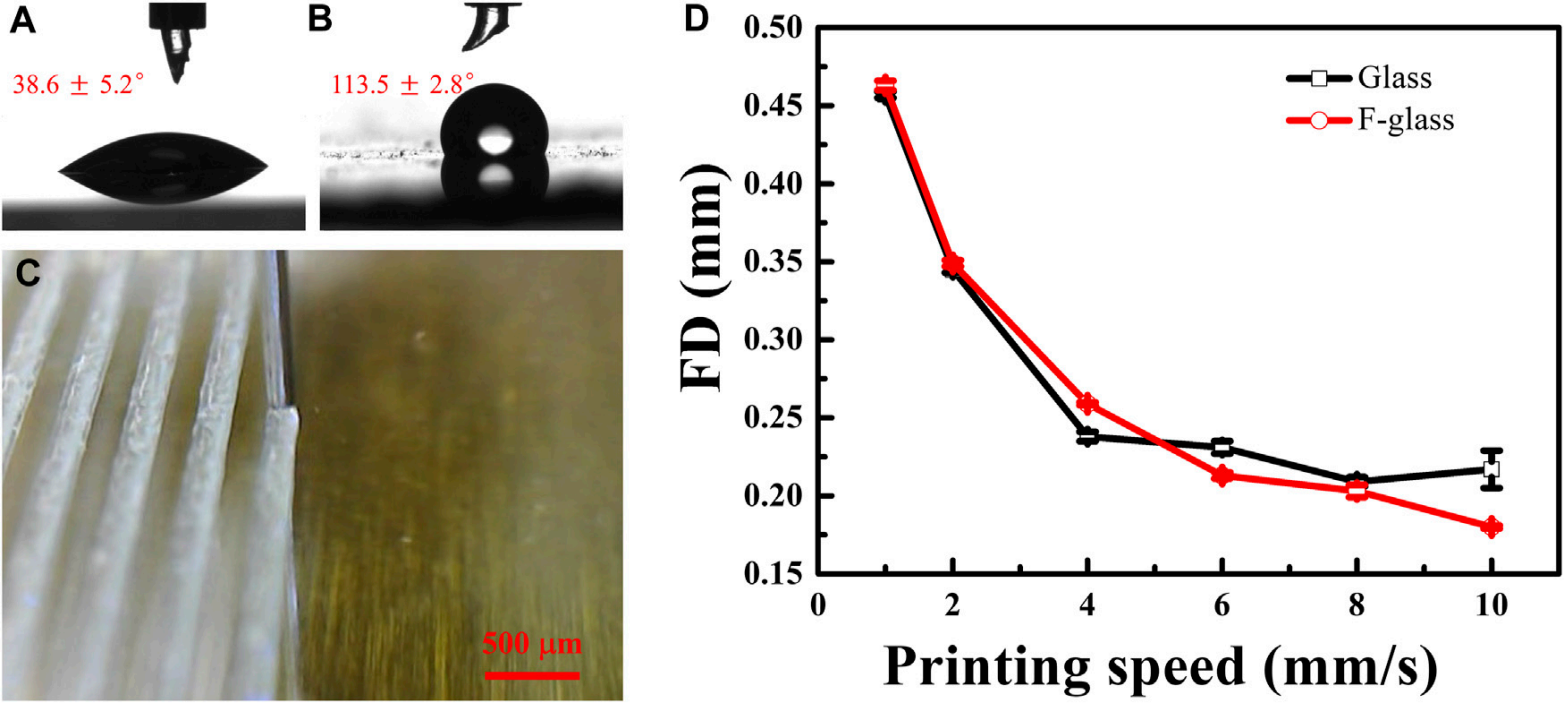
Water droplet profiles and WCAs on different glass substrates: **(A)** hydrophilic glass and **(B)** hydrophobic F-glass. **(C)** Optical image of the PDMS filament during its 3D printing on an F-glass substrate. **(D)** FD statistics of the PDMS filaments printed on glass and F-glass substrates at different printing speeds.

The hydrophilic glass substrate cleaned with distilled water and absolute ethyl alcohol in an ultrasonic cleaner for three times exhibited a WCA of 38.6° ± 5.2° (Figure 1A). The cleaned F-glass substrate obtained by PFTS treatment was hydrophobic and exhibited a WCA of 113.5° ± 2.8° (Figure 1B), thus indicating a lower surface energy, as expected. As shown in Figure 1C, although the PDMS filament was printed at a high printing speed of 8.00 mm/s on the hydrophobic F-glass substrate, the straightness, FD, and FS of the PDMS filaments were uniform during 3D printing. As demonstrated in our previous study (He et al., 2017), the printing speed had a notable effect on the FD statistics of the PDMS filaments. Therefore, the effect of the printing speed (1.00, 2.00, 4.00, 6.00, 8.00, and 10.00 mm/s) on the diameter of the PDMS filaments printed on the two types of glass substrates was also investigated. The FD statistics for the PDMS filaments printed on the hydrophilic glass and hydrophobic F-glass substrates at different printing speeds varied negligibly, except in the cases of the filaments printed with an extremely high printing speed of 10.00 mm/s (Figure 1D). The uniformity of the PDMS filament was almost unaffected by the printing speed or surface wettability of the glass substrate, as indicated by the small deviations in the FD values.

**FIGURE 2:**
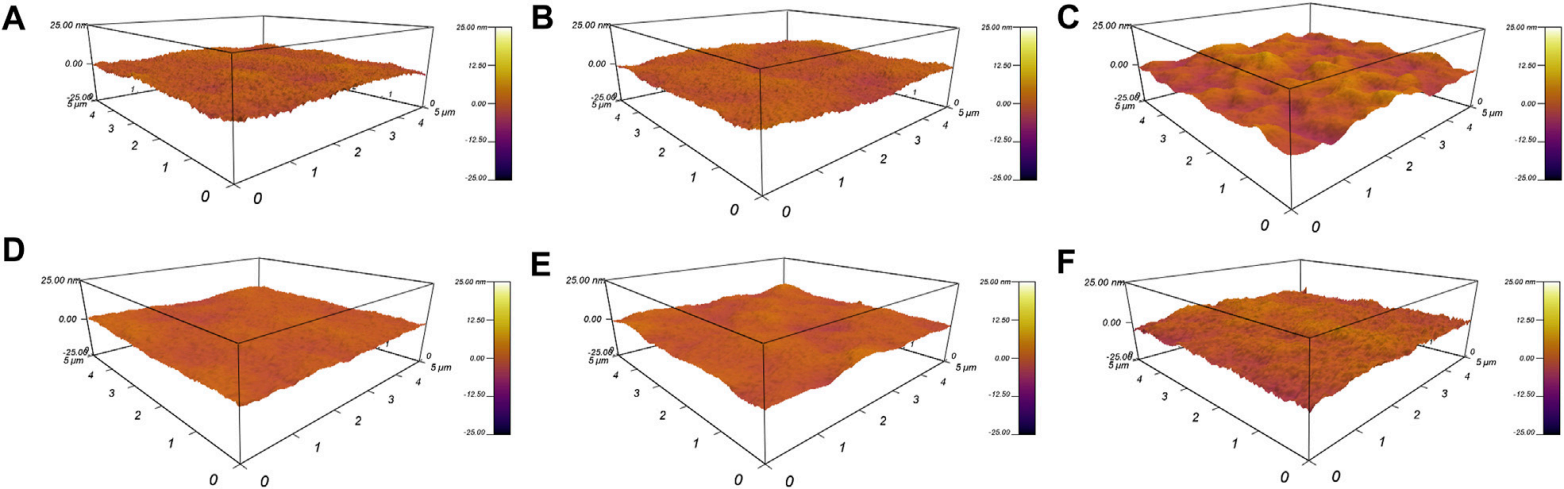
AFM images showing the surface morphologies of the PDMS filaments printed on **(A–C)** hydrophilic glass and **(D–F)** hydrophobic F-glass substrates at different printing speeds: **(A,D)** 1.00 mm/s; **(B,E)** 6.00 mm/s; **(C,F)** 10.00 mm/s.

**FIGURE 3:**
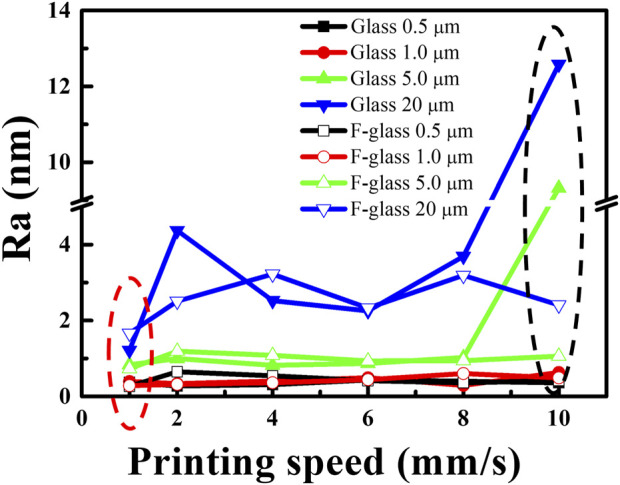
*R*a values of the PDMS filaments on hydrophilic glass and hydrophobic F-glass substrates for samples printed using different printing speeds.

In general, the FD decreased with increasing printing speed owing to the higher tensile force at a higher printing speed. When the printing speed was as high as 10.00 mm/s, the FD on the hydrophilic glass differed only slightly compared with that of the sample printed at 8.00 mm/s. However, on the hydrophobic F-glass, the FD obtained at a printing speed of 10.00 mm/s was still lower than that obtained at a printing speed of 8.00 mm/s. This phenomenon can be attributed to the large difference in the surface energy between the hydrophilic glass substrate and hydrophobic PDMS filament. This result indicates that the surface roughness of the PDMS filament on the hydrophilic glass may be greater than that on the hydrophobic F-glass. The AFM images of the PDMS filaments printed at different printing speeds (1.00, 6.00, and 10.00 mm/s) on hydrophilic glass (Figures 2A–C) and hydrophobic F-glass (Figures 2D–F) substrates demonstrate that both the printing speed and the surface wettability of the glass substrate can affect the surface morphology of the PDMS filament. Moreover, the quantitative *R*a values of the filaments over 0.5 × 0.5, 1.0 × 1.0, 5.0 × 5.0, and 20.0 mm × 20.0 mm areas were statistically evaluated to understand the effects of the printing speed and substrate surface wettability on the surface morphology of the PDMS filaments (Figure 3). For small statistical areas of 0.5 × 0.5 and 1.0 mm × 1.0 mm, the *R*a values of the samples obtained using different printing speeds differed negligibly, regardless of the substrate type. However, for larger statistical areas of 5.0 × 5.0 and 20.0 mm × 20.0 mm, the *R*a values of the different samples differed significantly. As the printing speed increased, the *R*a of the PDMS filament increased for both the hydrophilic and hydrophobic substrate cases. Therefore, a higher printing speed is expected to increase the surface roughness (black dashed circle in Figure 3), which might be the reason for the slight decrease in the FD on the hydrophilic glass when the printing speed was as high as 10.00 mm/s. Moreover, the *R*a of the PDMS filament on the hydrophilic glass was greater than that of the filament on the hydrophobic F-glass at different printing speeds. In addition, at a given printing speed, the *R*a of the PDMS filament on the hydrophobic F-glass was more controllable compared with the *R*a of the filament on the hydrophilic glass. Considering that a substrate with a lower surface energy is beneficial for peeling off the printed film, the hydrophobic F-glass and a low printing speed are more suitable for achieving better control over the FD and surface morphology of the PDMS filament (red dashed circle in Figure 3).

### 3.2 Effect of the number of printed layers on the filament morphology and wettability of the PDMS filament and porous PDMS film

**FIGURE 4:**
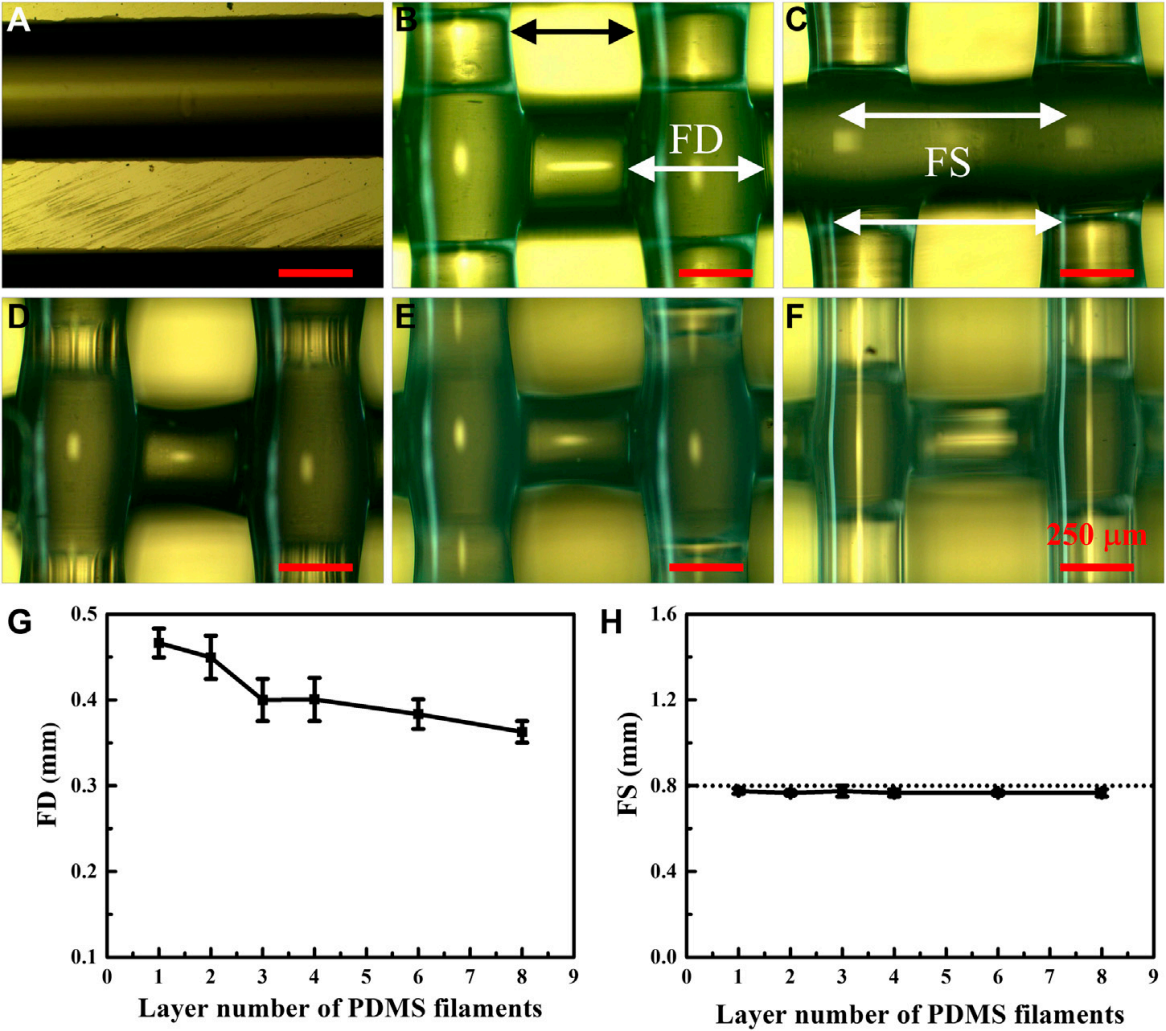
Optical images showing the surface morphologies of PDMS filaments in samples with different numbers of printed layers: **(A)** one, **(B)** two, **(C)** three, **(D)** four, **(E)** six, and **(F)** eight layers. **(G)** FD and **(H)** FS values of the PDMS filaments printed on hydrophobic F-glass substrates as a function of the layer number.

PDMS films with different numbers of filament layers (Xie et al., 2019; He et al., 2021a; He et al., 2021b; Eloffy et al., 2022; Eloffy et al., 2022; He et al., 2023a; He et al., 2023b) were 3D printed on hydrophobic F-glass substrates with a fixed FS of 0.8 mm along both X- and *Y*-axes at a printing speed of 0.75 mm/s, which is lower than the speed used to print the samples presented in Figure 1. The optical images in Figure 4A reveal that the PDMS filament in the single-layer film is straight and smooth, similar to that in the image in Figure 1C. However, the PDMS filaments in the multilayer films (Figures 4B–F) were deformed owing to downward tensile bending at nonoverlapping locations under the action of gravity (shown using a black double-headed arrow in Figure 4B). Consequently, the FD statistics of the PDMS filaments at different measured positions were not the same. Thus, the FDs reported here for films with two or more layers (Figure 4G) are the averaged data of five specific positions, as indicated by the white double-headed arrow in Figure 4B. The decreased statistical FD with an increase in the number of PDMS filament layers (Figure 4G) indicates that the number of printing layers affects the morphology of the individual PDMS filaments and, thus, the porous PDMS films composed of these filaments. Although the PDMS filaments in the multilayer samples were evidently deformed, the FS of the PDMS filaments was unaffected by their deformation and could be measured precisely at any position of the filament (indicated by white double-headed arrows in Figure 4C). Remarkably, the measured FS of the PDMS filaments was almost the same as the designed FS (0.8 mm), regardless of the number of layers (Figure 4H). Thus, the number of printing layers hardly affected the FS of the PDMS filaments, and the FS was primarily determined by the precision of the 3D printer (He et al., 2017).

**FIGURE 5:**
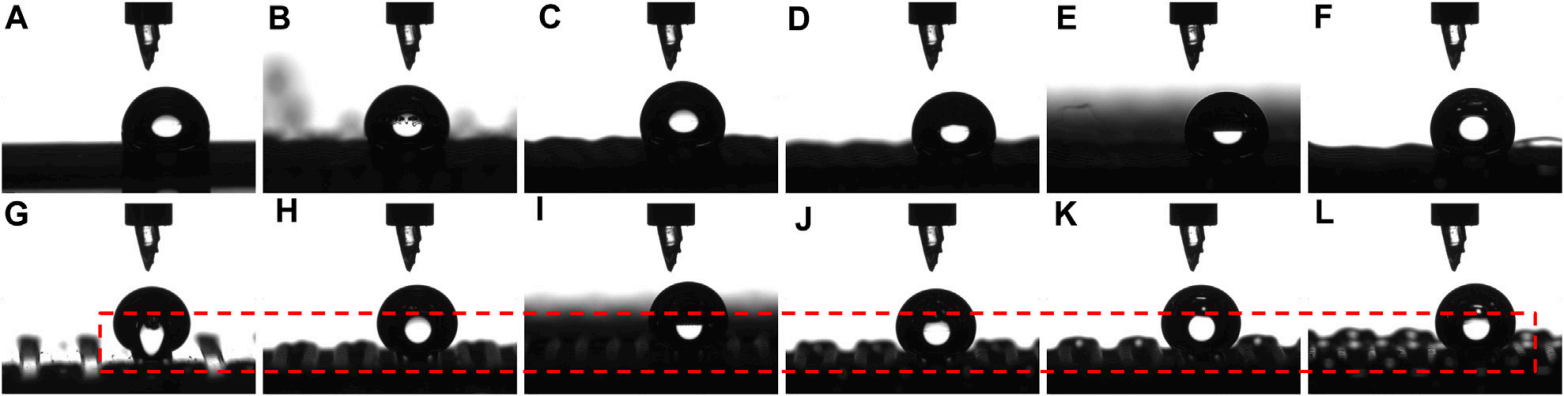
Water droplet profiles on PDMS surfaces in **(A–F)** parallel and **(G–L)** perpendicular directions for samples with different numbers of printing layers on hydrophobic F-glass substrates: **(A,G)** one; **(B,H)** two; **(C,I)** three; **(D,J)** four; **(E,K)** six; **(F,L)** eight.

**FIGURE 6:**
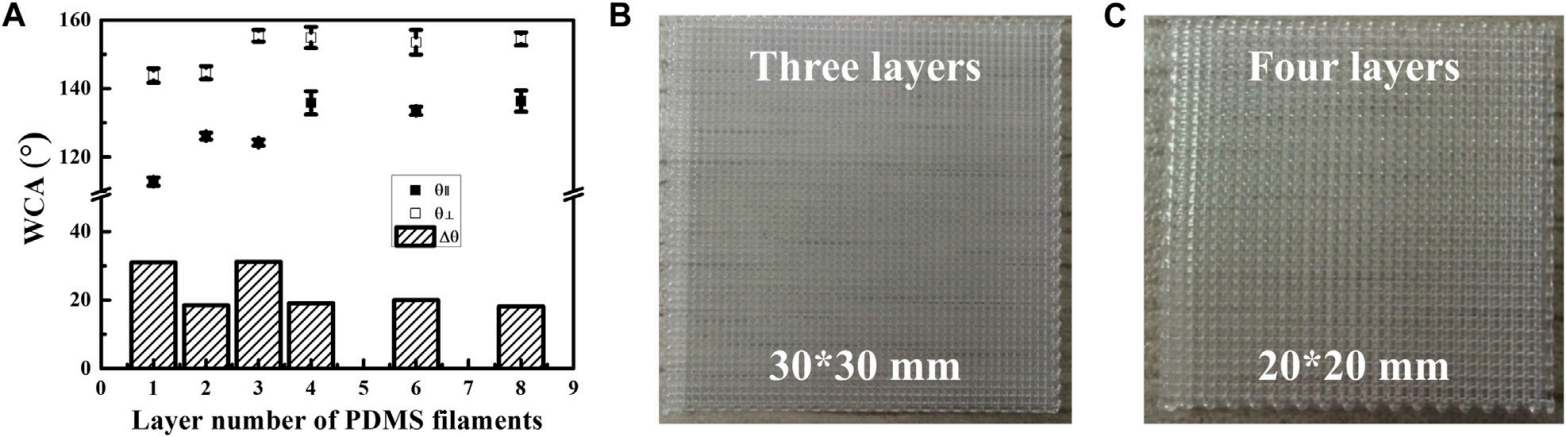
**(A)** WCAs and ASW of PDMS film surfaces in parallel and perpendicular directions for samples with different number of printed layers on hydrophobic F-glass substrates. Optical images of [**(B)**, 30 mm in length and width] larger-area 3D printed PDMS films with three layers and [**(C)**, 20 mm in length and width] four layers.

In general, the wettability of a surface is exclusively affected by the surface morphology when the chemical composition of the surface is fixed. All samples were fabricated using hydrophobic PDMS of the same chemical composition, which has been extensively characterized in our previous studies (He et al., 2017; Yu et al., 2018). Therefore, in this study, we focused on the effect of the surface morphology of the individual PDMS filament, which is affected by the number of printed layers, on the surface wettability of the PDMS film. As shown in Figure 5, the water droplet behavior in parallel (Figures 5A–F) and perpendicular (Figures 5G–L) directions was characterized for samples with one (Figures 5A, G), two (Figures 5B, H), three (Figures 5C, I), four (Figures 5D, J), six (Figures 5E, K), and eight (Figures 5F, L) printed layers of PDMS filaments. All film samples were hydrophobic and exhibited clear ASW characteristics. The averaged WCAs of different samples are presented in Figure 6A. When the number of printed layers was ≤4, the WCAs in both the parallel and perpendicular directions gradually increased with increasing number of printed layers. This phenomenon can be attributed to the following three factors: first, the deformation of the PDMS filaments in the multilayer samples increases the porosity of the PDMS film (Figures 4B–D). Second, the significant decrease in the FD (Figure 4G) further increases the porosity of the PDMS film. Based on the Cassie–Baxter model (Cassie and Baxter, 1944), an increased porosity of a sample can result in increased surface air fraction, thereby leading to improved surface hydrophobicity. Third, as the number of printed layers increases, the effect of the surface wettability of the glass substrate on the PDMS film decreases. According to the Wenzel model (Wenzel, 1936), because the glass substrate has a lower surface roughness, water droplets on the porous PDMS films with a lower number of printing layers are probably adsorbed by the smooth glass substrate, thereby resulting in a lower WCA. As shown by the red dashed rectangle in Figure 5, the adsorption behavior of water droplets located between the PDMS filaments in a single-layer film resulted in the deformation of the water droplet (Figure 5G), as confirmed by the observation of a white ellipse in the center of the water droplet profile, whereas the center of the droplet profile appeared as a white circle in the other cases. However, the WCAs of the samples with six and eight PDMS layers were almost identical to those of the samples with four layers. This result indicates that the final surface wettability of the PDMS film was hardly affected by the deformation of the PDMS filament (Figures 4E, F), the slight decrease in the FD (Figure 4G), and the surface wettability of the smooth glass substrate. Essentially, the surface wettability of the 3D printed PDMS film remains stable when the number of printing layers reaches four, as further demonstrated by the ASW results (Δθ) in Figure 6A.

In addition, larger-area 3D printed PDMS films with three (Figure 6B, 30 mm in length and width) and four (Figure 6C, 20 mm in length and width) layers were fabricated to investigate the effect of the number of printed layers on the macroscopic surface morphology of the film. The PDMS filaments in the films with three and four layers maintained their stability and regularity, and no evident defects were observed in the films. Thus, the number of printing layers had a negligible effect on the macroscopic surface morphology but a significant effect on the microscopic surface morphology of the 3D printed PDMS films.

### 3.3 Effect of the structural design of layers on the morphology and surface wettability of porous PDMS films

**FIGURE 7:**
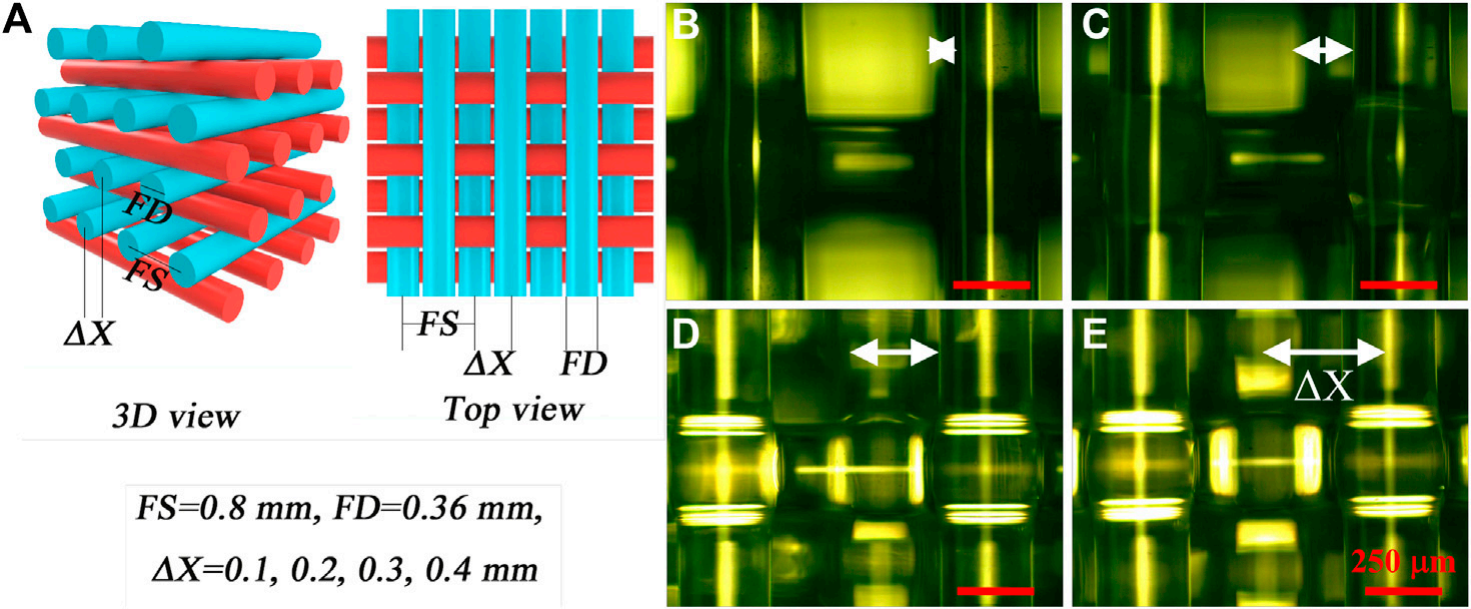
**(A)** Different structural designs of porous PDMS films with different shifted distances (ΔX) between the filaments in adjacent printed layers. **(B–E)** Optical images showing the surface morphologies of the PDMS films with different ΔX values: **(B)** 0.1; **(C)** 0.2; **(D)** 0.3; **(E)** 0.4 mm.

As is well known, the surface morphology of a substrate can significantly affect its wettability. Therefore, porous PDMS films with different structural arrangements in different layers were designed (Figure 7A), as they could be easily fabricated by 3D printing (Figures 7B–E). As shown in Figure 7A, the eight-layered samples with a certain shifted distance (a typical ΔX of 0.4 mm; see the illustration in Figure 7A) both in the X- and Y-axes were 3D printed on hydrophobic F-glass with an FS of 0.8 mm at a low speed of 0.75 mm/s (FD of approximately 0.36 mm). The PDMS filaments in layers 5–8 had the same arrangement as in layers 1–4. The shifted distances (ΔX) of the PDMS filaments in layers 3 and 7 (Xie et al., 2019; He et al., 2021b) were designed to be 0.1, 0.2, 0.3, or 0.4 mm with respect to the filaments in layers 1 and 5 (He et al., 2021a; Eloffy et al., 2022). The corresponding shifted distances (ΔX = 0.1, 0.2, 0.3, and 0.4 mm) are shown as white double-headed arrows in the optical images of the samples in Figures 7B–E. The FD and FS of the PDMS filaments with different shifted distances (ΔX) were similar to those of the sample in Figure 4F with no shifted distance. Thus, the various structural designs in this study had a negligible effect on the microscopic surface morphology of the PDMS filaments but a notable effect on the macroscopic surface morphology of the 3D printed porous PDMS film.

**FIGURE 8:**
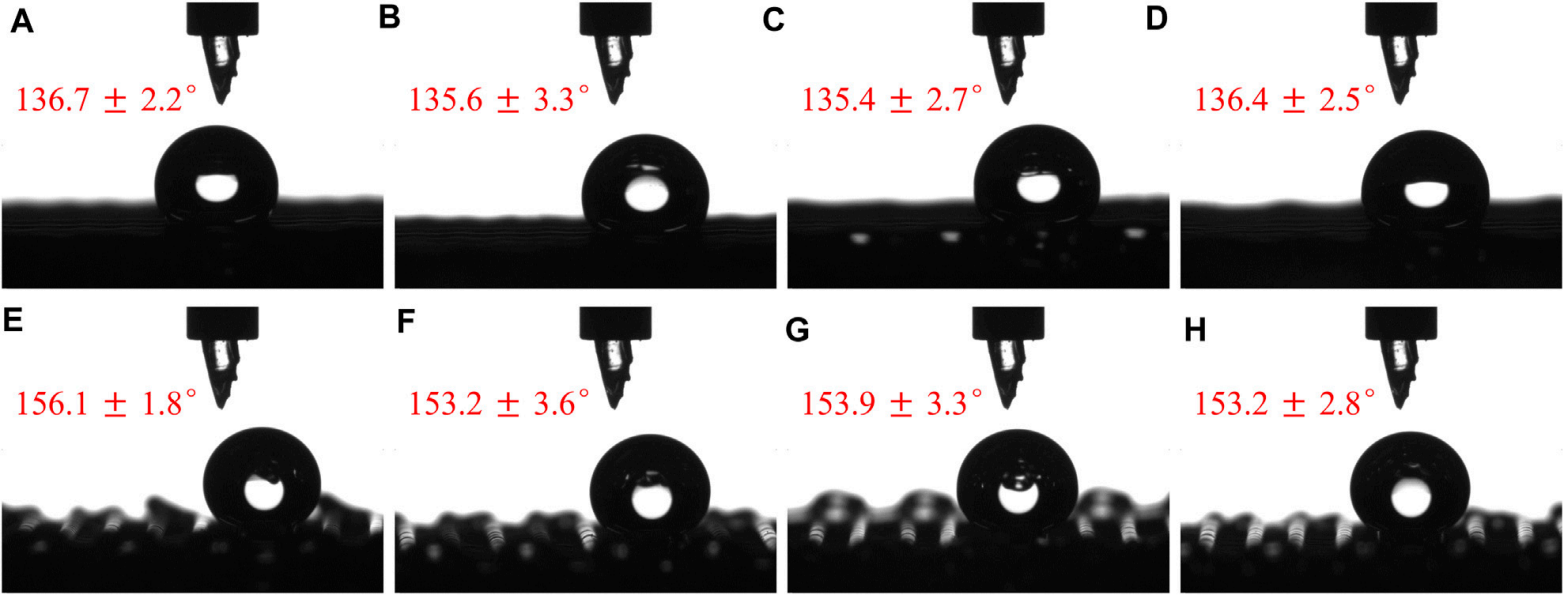
Water droplet profiles and WCAs on porous PDMS films with different ΔX in **(A–D)** parallel and **(E–H)** perpendicular directions. ΔX = **(A,E)** 0.1; **(B,F)** 0.2; **(C,G)** 0.3; **(D,H)** 0.4 mm.

In general, distinct regular macroscopic physical structures would result in different surface wettabilities. However, as shown in Figure 8, the WCAs on the 3D printed porous PDMS films with different shifted distances (ΔX) of 0.1 (Figures 8A, E), 0.2 (Figures 8B, F), 0.3 (Figures 8C, G), and 0.4 mm (Figures 8D, H) differed negligibly not only in the parallel direction (Figures 8A–D) but also in the perpendicular (Figures 8E–H) one. The WCAs of the samples with different shifted distances (ΔX) were similar to those in Figure 6A with no shifted distance. Therefore, the surface wettability of the 3D printed porous PDMS film was determined by the surface morphology of the top layer of the sample only and not on the shifted distance (ΔX). This surprising phenomenon of surface wettability makes the process versatile and various physical structural designs of 3D printed porous PDMS films with controllable and stable surface wettability can be prepared, such as those exhibiting ASW and superhydrophobicity, thus resulting in extensive applications in biomedical, environmental, and structural mechanical fields.

## 4 Conclusion

Hydrophilic glass and PFTS-treated hydrophobic glass (i.e., F-glass) substrates were used for the 3D printing of PDMS filaments. The wettability of the glass substrate hardly affected the FD in a single-layer film, except for the samples obtained with an extremely high printing speed of 10.00 mm/s. In general, the FD decreased with increasing printing speed. The surface roughness of the PDMS filament increased with increasing printing speed, regardless of the substrate type (hydrophilic or hydrophobic). However, the surface roughness of the PDMS film printed on the hydrophilic glass was greater than that on the hydrophobic F-glass at different printing speeds. Therefore, the hydrophobic F-glass and a lower printing speed were more suitable for achieving better control over the FD and surface morphology of the PDMS filament. In addition, the number of printed layers significantly affected the morphology of the PDMS filament and porous PDMS film and thus the surface wettability of the film. The filament was straight in the single-layer film but deformed in the multilayer film. Statistically, the FD decreased with an increase in the number of printing layers. However, the FS remained unaffected by the deformation of the PDMS filament or the number of printed layers. For films with four layers or less, the WCAs in both the parallel and perpendicular directions gradually increased with the number of printed layers. However, the WCAs of the samples with six and eight printed layers were almost the same as those of the sample with four layers. Structural designs with different shifted distances (ΔX) led to negligible differences between the FD and FS of the PDMS filaments. The WCAs of these samples also showed negligible differences in the parallel and perpendicular directions. Therefore, the surface wettability of the 3D printed porous PDMS films was determined by the surface morphology of the topmost layer, and it hardly varied with the shifted distance (ΔX). This study provides a novel strategy for producing 3D printed porous PDMS films of various physical structural designs that exhibit controllable and stable surface wettability, such as ASW and superhydrophobicity, which can be extensively applied in biomedical, environmental, and structural mechanical fields.

## Data Availability

The original contributions presented in the study are included in the article/Supplementary Material, further inquiries can be directed to the corresponding authors.

## References

[B1] Al-Bishari A. M. Al-Shaaobi B. A. Al-Bishari A. A. Al-Baadani M. A. Yu L. Shen J. (2022). Vitamin D and curcumin-loaded PCL nanofibrous for engineering osteogenesis and immunomodulatory scaffold. Front. Bioeng. Biotechnol. 10, 975431. 10.3389/fbioe.2022.975431 36003534 PMC9393239

[B2] Bliznyuk O. Vereshchagina E. Kooij E. S. Poelsema B. (2009). Scaling of anisotropic droplet shapes on chemically stripe-patterned surfaces. Phys. Rev. E 79 (4), 041601. 10.1103/physreve.79.041601 19518239

[B3] Bohn H. F. Federle W. (2004). Insect aquaplaning: nepenthes pitcher plants capture prey with the peristome, a fully wettable water-lubricated anisotropic surface. Proc. Natl. Acad. Sci. U. S. A. 101 (39), 14138–14143. 10.1073/pnas.0405885101 15383667 PMC521129

[B4] Cao X. Pan J. Cai G. Xiao S. Ma X. Zhang X. (2022). A chemically robust and self-healing superhydrophobic polybenzoxazine coating without fluorocarbon resin modification: fabrication and failure mechanism. Prog. Org. Coatings 163, 106630. 10.1016/j.porgcoat.2021.106630

[B5] Cassie A. B. D. Baxter S. (1944). Wettability of porous surfaces. Trans. Faraday Soc. 40, 546–551. 10.1039/tf9444000546

[B6] Chang J. He X. Yang Z. Bai X. Yuan C. (2022). Effects of chemical composition on the hydrophobicity and antifouling performance of epoxy-based self-stratifying nanocomposite coatings. Prog. Org. Coatings 167, 106827. 10.1016/j.porgcoat.2022.106827

[B7] Eloffy M. G. El-Sherif D. M. Abouzid M. Elkodous M. A. El-nakhas H. S. Sadek R. F. (2022). Proposed approaches for coronaviruses elimination from wastewater: membrane techniques and nanotechnology solutions. Nanotechnol. Rev. 11 (1), 1–25. 10.1515/ntrev-2022-0001

[B8] Feng L. Zhang Y. A. Xi J. M. Zhu Y. Wang N. Xia F. (2008). Petal effect: A superhydrophobic state with high adhesive force. Langmuir 24 (8), 4114–4119. 10.1021/la703821h 18312016

[B9] Ghahramani P. Behdinan K. Moradi-Dastjerdi R. Naguib H. E. (2022). Theoretical and experimental investigation of MWCNT dispersion effect on the elastic modulus of flexible PDMS/MWCNT nanocomposites. Nanotechnol. Rev. 11 (1), 55–64. 10.1515/ntrev-2022-0006

[B10] Gresham I. J. Neto C. (2023). Advances and challenges in slippery covalently-attached liquid surfaces. Adv. Colloid Interface Sci. 315, 102906. 10.1016/j.cis.2023.102906 37099851

[B11] Guo P. Wang Z. Han X. Heng L. (2021). Nepenthes pitcher inspired isotropic/anisotropic polymer solid–liquid composite interface: preparation, function, and application. Mater. Chem. Front. 5 (4), 1716–1742. 10.1039/D0QM00805B

[B12] Han X. Gong X. (2021). *In situ*, one-pot method to prepare robust superamphiphobic cotton fabrics for high buoyancy and good antifouling. ACS Appl. Mater. Interfaces 13 (26), 31298–31309. 10.1021/acsami.1c08844 34156810

[B13] He Z. Chen Y. Yang J. Tang C. Lv J. Liu Y. (2017). Fabrication of Polydimethylsiloxane films with special surface wettability by 3D printing. Compos. Part B Eng. 129, 58–65. 10.1016/j.compositesb.2017.07.025

[B14] He Z. Lan X. Hu Q. Li H. Li L. Mao J. (2021a). Antifouling strategies based on super-phobic polymer materials. Prog. Org. Coatings 157, 106285. 10.1016/j.porgcoat.2021.106285

[B15] He Z. Ma M. Lan X. Chen F. Wang K. Deng H. (2011). Fabrication of a transparent superamphiphobic coating with improved stability. Soft Matter 7 (14), 6435–6443. 10.1039/c1sm05574g

[B16] He Z. Ma M. Xu X. Wang J. Chen F. Deng H. (2012). Fabrication of superhydrophobic coating via a facile and versatile method based on nanoparticle aggregates. Appl. Surf. Sci. 258 (7), 2544–2550. 10.1016/j.apsusc.2011.10.090

[B17] He Z. Mu L. Wang N. Su J. Wang Z. Luo M. (2023a). Design, fabrication, and applications of bioinspired slippery surfaces. Adv. Colloid Interface Sci. 318, 102948. 10.1016/j.cis.2023.102948 37331090

[B18] He Z. Rabiee N. Wei Q. Hou Y. Yan B. Xie J. (2023b). Editorial: composites and surface and interface engineering (CSIE): preparation and modification of biomaterials and their anti-biofouling ability and surface wettability. Front. Bioeng. Biotechnol. 11, 1230571. 10.3389/fbioe.2023.1230571 37378044 PMC10292077

[B19] He Z. Wang N. Yang X. Mu L. Wang Z. Su J. (2023c). Antifouling induced by surface wettability of poly(dimethyl siloxane) and its nanocomposites. Nanotechnol. Rev. 12 (1). 10.1515/ntrev-2022-0552

[B20] He Z. Yang X. Mu L. Wang N. Lan X. (2022). A versatile “3M” methodology to obtain superhydrophobic PDMS-based materials for antifouling applications. Front. Bioeng. Biotechnol. 10, 998852. 10.3389/fbioe.2022.998852 36105602 PMC9464926

[B21] He Z. Yang X. Wang N. Mu L. Pan J. Lan X. (2021b). Anti-Biofouling polymers with special surface wettability for biomedical applications. Front. Bioeng. Biotechnol. 9 (1260), 807357. 10.3389/fbioe.2021.807357 34950651 PMC8688920

[B22] Ji Z. Liu Y. Du F. (2021). Rational design of superhydrophobic, transparent hybrid coating with superior durability. Prog. Org. Coatings 157, 106294. 10.1016/j.porgcoat.2021.106294

[B23] Jiang T. Guo Z. Liu W. (2015). Biomimetic superoleophobic surfaces: focusing on their fabrication and applications. J. Mater. Chem. A 3 (5), 1811–1827. 10.1039/C4TA05582A

[B24] Kusumaatmaja H. Vrancken R. J. Bastiaansen C. W. M. Yeomans J. M. (2008). Anisotropic drop morphologies on corrugated surfaces. Langmuir 24 (14), 7299–7308. 10.1021/la800649a 18547090

[B25] Li L. Chen C. Zhang C. Luo R. Lan X. Guo F. (2021). A honokiol-mediated robust coating for blood-contacting devices with anti-inflammatory, antibacterial and antithrombotic properties. J. Mater. Chem. B 9 (47), 9770–9783. 10.1039/D1TB01617B 34806726

[B26] Li L. Liu L. Li L. Guo F. Ma L. Fu P. (2022b). Chitosan coated bacteria responsive metal-polyphenol coating as efficient platform for wound healing. Compos. Part B Eng. 234, 109665. 10.1016/j.compositesb.2022.109665

[B27] Li L. Wang Y. Liu K. Yang L. Zhang B. Luo Q. (2022a). Nanoparticles-stacked superhydrophilic coating supported synergistic antimicrobial ability for enhanced wound healing. Mater. Sci. Eng. C 132, 112535. 10.1016/j.msec.2021.112535 35090805

[B28] Li L. Xu Z. Sun W. Chen J. Dai C. Yan B. (2020a). Bio-inspired membrane with adaptable wettability for smart oil/water separation. J. Membr. Sci. 598, 117661. 10.1016/j.memsci.2019.117661

[B29] Li L. Yang L. Liao Y. Yu H. Liang Z. Zhang B. (2020b). Superhydrophilic versus normal polydopamine coating: A superior and robust platform for synergistic antibacterial and antithrombotic properties. Chem. Eng. J. 402, 126196. 10.1016/j.cej.2020.126196

[B30] Li Y. Jin D. Fan Y. Zhang K. Yang T. Zou C. (2023). Preparation and performance of random- and oriented-fiber membranes with core–shell structures via coaxial electrospinning. Front. Bioeng. Biotechnol. 10, 1114034. 10.3389/fbioe.2022.1114034 36698642 PMC9868300

[B31] Liao Y. Chen X. Jiang Y. Qu C. Liu X. Zhao A. (2023). Piranha solution treatment: A facile method for improving the antithrombotic ability and regulating smooth muscle cell growth on blood contact materials. Front. Bioeng. Biotechnol. 11, 1166334. 10.3389/fbioe.2023.1166334 36994360 PMC10040745

[B32] Luo Q. Peng J. Chen X. Zhang H. Deng X. Jin S. (2022). Recent advances in multifunctional mechanical–chemical superhydrophobic materials. Front. Bioeng. Biotechnol. 10, 947327. 10.3389/fbioe.2022.947327 35910015 PMC9326238

[B33] Mallakpour S. Tabesh F. Hussain C. M. (2021b). 3D and 4D printing: from innovation to evolution. Adv. Colloid Interface Sci. 294, 102482. 10.1016/j.cis.2021.102482 34274721

[B34] Mallakpour S. Tabesh F. Hussain C. M. (2022). A new trend of using poly(vinyl alcohol) in 3D and 4D printing technologies: process and applications. Adv. Colloid Interface Sci. 301, 102605. 10.1016/j.cis.2022.102605 35144173

[B35] Mallakpour S. Tukhani M. Hussain C. M. (2021a). Recent advancements in 3D bioprinting technology of carboxymethyl cellulose-based hydrogels: utilization in tissue engineering. Adv. Colloid Interface Sci. 292, 102415. 10.1016/j.cis.2021.102415 33892215

[B36] Marovič N. Ban I. Maver U. Maver T. (2023). Magnetic nanoparticles in 3D-printed scaffolds for biomedical applications. Nanotechnol. Rev. 12 (1). 10.1515/ntrev-2022-0570

[B37] Morita M. Koga T. Otsuka H. Takahara A. (2005). Macroscopic-wetting anisotropy on the line-patterned surface of fluoroalkylsilane monolayers. Langmuir 21 (3), 911–918. 10.1021/la0485172 15667167

[B38] Pan Y. Liu Y. Yang S. Zhang C. Ullah Z. (2023). Recent research progress on the stimuli-responsive smart membrane: A review. Nanotechnol. Rev. 12 (1). 10.1515/ntrev-2022-0538

[B39] Rasitha T. P. Vanithakumari S. C. Nanda Gopala Krishna D. George R. P. Srinivasan R. Philip J. (2022). Facile fabrication of robust superhydrophobic aluminum surfaces with enhanced corrosion protection and antifouling properties. Prog. Org. Coatings 162, 106560. 10.1016/j.porgcoat.2021.106560

[B40] Rosenberg R. (2005). Why is ice slippery? Phys. Today 58 (12), 50–54. 10.1063/1.2169444

[B41] Saji V. S. (2021). Carbon nanostructure-based superhydrophobic surfaces and coatings. Nanotechnol. Rev. 10 (1), 518–571. 10.1515/ntrev-2021-0039

[B42] Selim M. S. Fatthallah N. A. Higazy S. A. Hao Z. Jing Mo P. (2022). A comparative study between two novel silicone/graphene-based nanostructured surfaces for maritime antifouling. J. Colloid Interface Sci. 606, 367–383. 10.1016/j.jcis.2021.08.026 34392032

[B43] Selim M. S. Azzam A. M. Higazy S. A. El-Safty S. A. Shenashen M. A. (2022). Novel graphene-based ternary nanocomposite coatings as ecofriendly antifouling brush surfaces. Prog. Org. Coatings 167, 106803. 10.1016/j.porgcoat.2022.106803

[B44] Seo E. Park J. Gil J.-E. Lim H. Lee D. Lee S. J. (2021). Multifunctional biopolymer coatings inspired by loach skin. Prog. Org. Coatings 158, 106383. 10.1016/j.porgcoat.2021.106383

[B45] Shinde A. Siva I. Munde Y. Sankar I. Sultan M. T. H. Shahar F. S. (2023). Appraising the dielectric properties and the effectiveness of electromagnetic shielding of graphene reinforced silicone rubber nanocomposite. Nanotechnol. Rev. 12 (1). 10.1515/ntrev-2022-0558

[B46] Song P. Liu B. Liang C. Ruan K. Qiu H. Ma Z. (2021). Lightweight, flexible cellulose-derived carbon Aerogel@Reduced graphene oxide/PDMS composites with outstanding EMI shielding performances and excellent thermal conductivities. Nano-Micro Lett. 13, 91. 10.1007/s40820-021-00624-4 PMC8006522 34138335

[B47] Wang S. Feng D. Guan H. Guo Y. Liu X. Yan C. (2022b). Highly efficient thermal conductivity of polydimethylsiloxane composites via introducing “Line-Plane”-like hetero-structured fillers. Compos. Part A Appl. Sci. Manuf. 157, 106911. 10.1016/j.compositesa.2022.106911

[B48] Wang X. Huang J. Guo Z. (2022a). Overview of the development of slippery surfaces: lubricants from presence to absence. Adv. Colloid Interface Sci. 301, 102602. 10.1016/j.cis.2022.102602 35085985

[B49] Wang X. Wang Z. Heng L. Jiang L. (2020). Stable omniphobic anisotropic covalently grafted slippery surfaces for directional transportation of drops and bubbles. Adv. Funct. Mater. 30 (1). 10.1002/adfm.201902686

[B50] Wenzel R. N. (1936). Resistance of solid surfaces to wetting by water. Industrial Eng. Chem. 28, 988–994. 10.1021/ie50320a024

[B51] Wu D. Chen Q. D. Yao J. Guan Y. C. Wang J. N. Niu L. G. (2010). A simple strategy to realize biomimetic surfaces with controlled anisotropic wetting. Appl. Phys. Lett. 96 (5), 053704. 10.1063/1.3297881

[B52] Wu H. Zhang R. Sun Y. Lin D. D. Sun Z. Q. Pan W. (2008). Biomimetic nanofiber patterns with controlled wettability. Soft Matter 4 (12), 2429–2433. 10.1039/b805570j

[B53] Wu Y. Zeng J. Si Y. Chen M. Wu L. (2018). Large-area preparation of robust and transparent superomniphobic polymer films. ACS Nano 12 (10), 10338–10346. 10.1021/acsnano.8b05600 30299933

[B54] Xia D. Johnson L. M. López G. P. (2012). Anisotropic wetting surfaces with one-dimesional and directional structures: fabrication approaches, wetting properties and potential applications. Adv. Mater. 24 (10), 1287–1302. 10.1002/adma.201104618 22318857

[B55] Xie L. Gong L. Zhang J. Han L. Xiang L. Chen J. (2019). A wet adhesion strategy via synergistic cation–π and hydrogen bonding interactions of antifouling zwitterions and mussel-inspired binding moieties. J. Mater. Chem. A 7 (38), 21944–21952. 10.1039/c9ta08152f

[B56] Xu X. He Z. Wang Q. Chen F. Fu Q. (2015). Self-assembly of PS-b-PDMS on a tunable PDMS template with nanoscale channels and enhanced anisotropic wetting. Langmuir 31 (16), 4605–4611. 10.1021/acs.langmuir.5b00340 25844896

[B57] Yang C. Zeng Q. Huang J. Guo Z. (2022). Droplet manipulation on superhydrophobic surfaces based on external stimulation: A review. Adv. Colloid Interface Sci. 306, 102724. 10.1016/j.cis.2022.102724 35780752

[B58] Yang L. Li L. Wu H. Zhang B. Luo R. Wang Y. (2020). Catechol-mediated and copper-incorporated multilayer coating: an endothelium-mimetic approach for blood-contacting devices. J. Control. Release 321, 59–70. 10.1016/j.jconrel.2020.02.002 32035196

[B59] Yao W. Wu L. Sun L. Jiang B. Pan F. (2022). Recent developments in slippery liquid-infused porous surface. Prog. Org. Coatings 166, 106806. 10.1016/j.porgcoat.2022.106806

[B60] Young T. (1805). An essay on the cohesion of fluids. Philosophical Trans. R. Soc. Lond. 95 (0), 65–87. 10.1098/rstl.1805.0005

[B61] Yu H. Wu M. Duan G. Gong X. (2022). One-step fabrication of eco-friendly superhydrophobic fabrics for high-efficiency oil/water separation and oil spill cleanup. Nanoscale 14 (4), 1296–1309. 10.1039/D1NR07111D 35006232

[B62] Yu Y. Shao H. He Z. Tang C. Yang J. Li Y. (2018). Patternable poly(chloro-p-xylylene) film with tunable surface wettability prepared by temperature and humidity treatment on a polydimethylsiloxane/silica coating. Materials 11 (4), 486. 10.3390/ma11040486 29570696 PMC5951332

[B63] Zhang F. Xu D. Zhang D. Ma L. Wang J. Huang Y. (2021). A durable and photothermal superhydrophobic coating with entwinned CNTs-SiO2 hybrids for anti-icing applications. Chem. Eng. J. 423, 130238. 10.1016/j.cej.2021.130238

[B64] Zhang J. H. Sheng X. L. Jiang L. (2009). The dewetting properties of Lotus leaves. Langmuir 25 (3), 1371–1376. 10.1021/la8024233 19170641

[B65] Zhang L. Uzoma P. C. Xiaoyang C. Penkov O. V. Hu H. (2022). Bio-Inspired hierarchical micro/nanostructured surfaces for superhydrophobic and anti-ice applications. Front. Bioeng. Biotechnol. 10, 872268. 10.3389/fbioe.2022.872268 35387304 PMC8977784

[B66] Zhang M. Wang L. Hou Y. Shi W. Feng S. Zheng Y. (2015). Controlled smart anisotropic unidirectional spreading of droplet on a fibrous surface. Adv. Mater. 27 (34), 5057–5062. 10.1002/adma.201502143 26198463

[B67] Zhang T. Li M. Su B. Ye C. Li K. Shen W. (2011). Bio-inspired anisotropic micro/nano-surface from a natural stamp: grasshopper wings. Soft Matter 7 (18), 7973. 10.1039/c1sm05366c

[B68] Zhao H. Gao W.-C. Li Q. Khan M. R. Hu G.-H. Liu Y. (2022). Recent advances in superhydrophobic polyurethane: preparations and applications. Adv. Colloid Interface Sci. 303, 102644. 10.1016/j.cis.2022.102644 35313189

[B69] Zhao H. Law K.-Y. (2012). Directional self-cleaning superoleophobic surface. Langmuir 28 (32), 11812–11818. 10.1021/la301894e 22803516

[B70] Zhu Y. Chen M. Wu L. (2021). Synthesis of UV-responsive dual-functional microspheres for highly efficient self-healing coatings. Chem. Eng. J. 422, 130034. 10.1016/j.cej.2021.130034

